# ECG electrode localization using 3D visual reconstruction

**DOI:** 10.3389/fphys.2025.1504319

**Published:** 2025-03-12

**Authors:** Ayoub El Ghebouli, Amaël Mombereau, Michel Haïssaguerre, Rémi Dubois, Laura R. Bear

**Affiliations:** ^1^ University Bordeaux, Institut national de la sante et de la recherche medicale (INSERM), U-1045, IHU Liryc, Le Centre de Recherche Cardio-Thoracique de Bordeaux (CRCTB), Bordeaux, France; ^2^ CHU de Bordeaux, Cardiology-Electrophysiology and Stimulation Department, Institut national de la sante et de la recherche medicale (INSERM), U-1045, Bordeaux, France

**Keywords:** BSPM, ECG electrodes localization, 3D camera, 2D camera, AI

## Abstract

Body surface potential maps (BSPMs) derived from multi-channel ECG recordings enable the detection and diagnosis of electrophysiological phenomena beyond the standard 12-lead ECG. In this work, we developed two AI-based methods for the automatic detection of location of the electrodes used for BSPM: a rapid method using a specialized 3D Depth Sensing (DS) camera and a slower method that can use any 2D camera. Both methods were validated on a phantom model and in 7 healthy volunteers. With the phantom model, both 3D DS camera and 2D camera method achieved an average localization error less than 2 mm when compared to CT-scan or an Electromagnetic Tracking System (ETS). With healthy volunteers, the 3D camera yielded average 3D Euclidean distances ranging from 2.61 ± 1.2 mm to 5.78 ± 3.09 mm depending on the patient, similar to that seen with 2D camera (ranging from 2.45 ± 1.32 mm to 5.88 ± 2.73 mm). These results demonstrate high accuracy and provide practical alternatives to traditional imaging techniques, potentially enhancing the interest of BSPMs in a clinical setting.

## 1 Introduction

Body surface potential mapping (BSPM) uses multi-channel ECG recordings (up to 256 electrodes) to record from broad areas and visualize the distribution of potentials temporally on three-dimensional (3-D) maps (Rodrigo et al., 2014; Gage et al., 2016; Ben Johnson et al., 2016). This enables the detection and potential diagnosis of electrophysiological phenomena outside the regions explored with the standard 12-lead ECG. Accurate acquisition of the ECG electrode locations on the body surface is often needed to minimize errors in the computational procedures that use BSPM. For example, BSPM can be used to reconstruct the electrical activity at the heart surface through the inverse problem of electrocardiography (Rudy, 2013; Haissaguerre et al., 2014; Bear et al., 2015), also known as electrocardiographic imaging (ECGI). This process requires accurate 3D locations of the electrodes aligned to an anatomical model of the patient’s thorax and heart.

To localize the electrodes in a clinical environment, non-contrast computed tomography (CT) is the simplest and most accurate way of acquiring 3D electrode locations. However, this type of imaging is associated with some radiation exposure, making it difficult to use in certain patient populations including healthy volunteers in clinical studies. Another option is magnetic resonance imaging (MRI), which unlike CT does not use ionizing radiation. MRI is not compatible with certain metallic hardware, limiting the acquisition system to specific MRI-opaque body surface electrodes and increases the cost of the procedure. Furthermore, the MRI requires additional time in the bore compared to CT, producing unnecessary discomfort from breath-holds for the patient.

An alternative approach is to use 3D visual reconstruction. Such systems offer the benefits of being low-cost, fast, and safe for the patient. Several research groups have proposed fully or semi-automatic 3D visual reconstruction systems for BSPM electrode positioning (Schulze et al., 2014; Perez-Alday et al., 2018; Alioui et al., 2017; Bayer et al., 2023; Shenoy et al., 2024). Early studies using these systems relied on stereo-photography, aligning two or more images of the electrodes from different angles to compute their 3D coordinates (Schulze et al., 2014; Ghanem et al., 2003). However, this approach can be time-consuming due to the need for calibration of the cameras and their alignment for each patient. The advent of 3D depth sensing cameras has addressed this limitation, enabling accurate localization of BSPM electrode positions using various camera models (Perez-Alday et al., 2018; Alioui et al., 2017; Bayer et al., 2023; Shenoy et al., 2024). Nonetheless, these systems still require specific 3D depth sensing cameras to be used. Furthermore, all previously reported methods (using 2D or 3D cameras) depend on either manually selecting electrode locations from images or attaching specific markers to the electrodes for their automatic detection.

This paper presents two novel fully automated deep learning-based methods for localizing ECG electrode positions. The first uses a 3D depth sensing (DS) camera, enabling near real-time automatic ECG electrode detection and labeling without additional markers, thus offering rapid results. The second method, while slower, utilizes any 2D video camera and does not require prior calibration, making it more accessible to various clinical setting. By incorporating both methods, we validate the robustness of our results and demonstrate their complementary advantages. Both methods yield similar accuracy in localizing ECG electrodes, allowing practitioners to choose an approach based on their specific needs, prioritizing speed with the 3D method or accessibility with the 2D method. Furthermore, the findings from this study open avenues for future research, particularly regarding the application of 2D video cameras in ECG mapping, as this approach has not been extensively explored in the literature.

These advancements hold significant potential to enhance clinical practice by making advanced ECG mapping more accessible, particularly in situations where traditional imaging methods are impractical or contraindicated. Both methods have been validated through phantom and human volunteer studies by comparing the electrode locations to those obtained through CT and an electromagnetic tracking system (ETS).

## 2 Materials and methods

**FIGURE 1:**
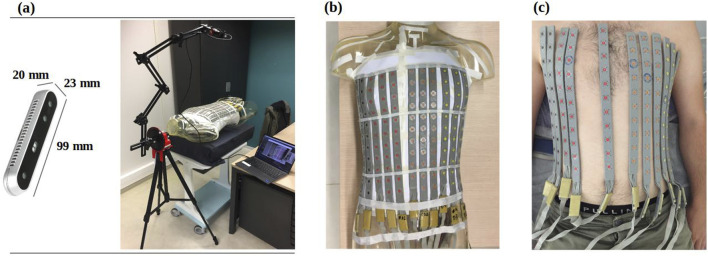
**(A)** RealSense D415 camera (left) mounted on an articulated arm to take photos from 12 fixed locations around the torso (right) **(B)** BioSemi strip electrodes fixed to a phantom torso and **(C)** on a healthy male volunteer.

A fully automated system for 3D ECG electrode localization based on 3D visual reconstruction was developed. Two types of cameras were employed, 1) an Intel RealSense Depth Camera D415 (Figure 1A) fixed in an articulated arm attached to a tripod to allow the camera to make a half rotation around the torso and 2) a smartphone camera to record a video by hand around the torso. The system was designed to localize BioSemi strip electrodes (BioSemi, the Netherlands) as shown in Figures 1B, C on the phantom and a human torso. The diameter of each electrode is 5 mm, with a 30 mm distance between two electrodes in the same strip. The following sections describe the methods used to automatically extract the 3D electrode locations using each camera (Section 2.1) and the methods used to validate their accuracy (Section 2.2).

### 2.1 Automatic ECG electrode localization using 3D visual reconstruction

**FIGURE 2:**
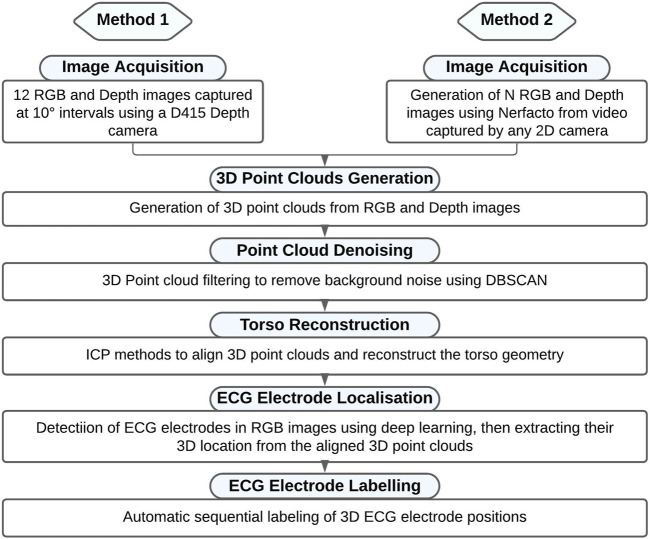
Flowchart outlining the 3D electrode localization and labelling process.

Figure 2 outlines the general pipeline for localizing and labelling the electrodes using the two different cameras to capture the initial images. A detailed explanation of each step is provided below.

#### 2.1.1 Image acquisition and 3D point cloud generation

**FIGURE 3:**
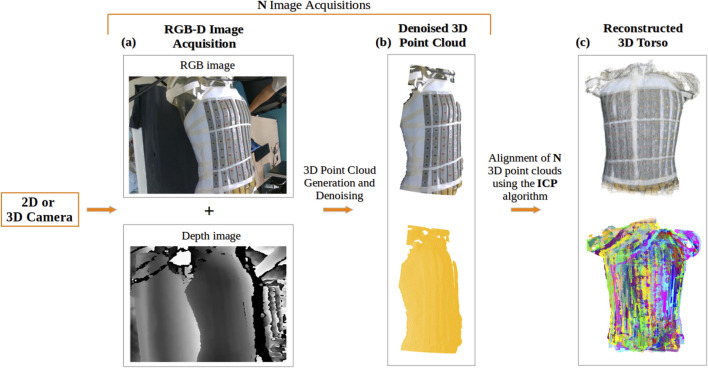
Reconstruction of the torso using a 2D or 3D camera: **(A)** Acquisition of RGB and depth images; **(B)** Generated 3D point cloud from a single acquisition of the torso, denoised using DBSCAN, shown in true color (top) and in yellow (bottom); **(C)** Final 3D torso point cloud resulting from the alignment of N acquisitions of the torso using the ICP-based algorithm, displayed in true color (top) and with distinct colors representing each view of the torso used in the alignment (bottom).

Two methods were employed to acquire the RGB-D (color and depth) images (Figure 3A).

##### 2.1.1.1 Method 1 (3D camera)

Using the Intel RealSense Depth Camera D415, 12 RGB-D images at a distance of approximately 60 cm from the torso were recorded, with a rotation of 10° between consecutive shots. With the 12 RGB-D images and camera’s intrinsic parameters, 12 3D point clouds were generated.

##### 2.1.1.2 Method 2 (2D camera)

Using a smartphone camera, a video of the torso was recorded by hand. Depth images corresponding to the RGB images of the video were predicted, along with the camera’s intrinsic parameters using Nerfacto (Tancik et al., 2023), a deep neural network architecture designed to process a collection of photographs captured from various angles of a specific scene, generating a volumetric representation of the scene. This enables the creation of a 3D point cloud for each frame in the video, resulting in N point clouds corresponding to the number of frames.

Following the acquisition of RGB-D images and creation of the corresponding point clouds, these point clouds were cleaned to remove noise.

#### 2.1.2 Point cloud denoising

For both methods, the generated point clouds, include information about the surrounding environment that can be considered noise. A density based clustering algorithm, DBSCAN (Density Based Spatial Clustering of Applications with Noise) (Ester et al., 1996), was used to filter the point clouds by selecting the largest region clustered (Figure 3B).

#### 2.1.3 Torso reconstruction

To achieve a complete 3D reconstruction of the torso (Figures 3C), the cleaned point clouds were aligned using the Iterative Closest Point (ICP) algorithm (Rusinkiewicz and Levoy, 2001), which computes the transformation matrix by minimizing the distance between two point clouds. We employed a sequential combination of two ICP variants: first, Point-to-Plane ICP, which minimizes distances to surface planes, followed by Point-to-Point ICP, which focuses on minimizing distances between corresponding points. To ensure reliable alignment throughout this process, we applied various distance thresholds to exclude irrelevant point pairs.

#### 2.1.4 ECG electrode localization

**FIGURE 4:**
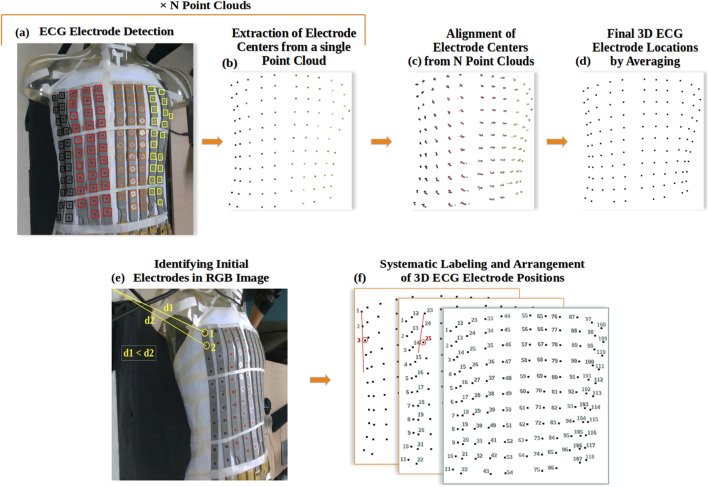
ECG Electrode localization: **(A)** Example of YOLOv8 detection and classification of ECG electrodes in RGB image, where black is for class A, red for B, orange for C, and yellow for D; **(B)** Extraction of electrode locations as a 3D point cloud; **(C)** Alignment of all electrode point clouds; **(D)** Final 3D reconstruction of ECG electrodes; **(E)** Automated identification of the initial and secondary ECG electrodes in the RGB image; **(F)** Systematic Labeling and Arrangement of 3D ECG Electrode Positions.

The ECG electrodes were separated into four classes based on their original color (A - brown, B - red, C - orange and D - yellow). To locate the electrodes in the point clouds, we followed a two-step approach. First, we used the YOLOv8 deep learning model (Jocher et al., 2023) to detect electrodes in the RGB images (Figure 4A). Then we extracted the centers of the detected regions in the point clouds (Figure 4B) to define the 3D electrode locations.

##### 2.1.4.1 ECG electrodes detection

The pre-trained YOLOv8 model (X-large architecture) (Jocher et al., 2023) was re-trained on a manually labeled dataset of ECG electrodes, comprising 160 JPG images that contained a total of 7,325 electrodes, with 19 images reserved for validation. The dataset included images of varying sizes to enhance the model’s adaptability. The model demonstrated high precision and recall across all classes, achieving an overall average precision (AP) of 98.8% on a test set of 75 images. Additionally, the area under the curve (AUC) scores derived from the receiver operating characteristic (ROC) curve for this test set were 98% for Classes A and D, and 97% for Classes B and C. These results underscore the model’s effectiveness in detecting ECG electrodes.

##### 2.1.4.2 ECG electrodes extraction from point clouds

Following the detection of electrodes in the N RGB images using YOLOv8, we extracted and averaged each detected region from the depth images, and then from the point clouds (Figure 4B). Using the transformation matrices obtained from the 3D reconstruction of the torso, we aligned the N 3D point clouds representing the electrodes (Figure 4C) and averaged each group of electrode points to achieve a complete 3D reconstruction of the ECG electrodes (Figure 4D), ensuring their precise 3D localization.

#### 2.1.5 ECG electrode labelling

To label the channel that each electrode corresponds to, we have developed an algorithm that systematically arranges the 3D electrode positions in sequential order, starting from the first electrode in class A to the last electrode in class D.

**FIGURE 5:**
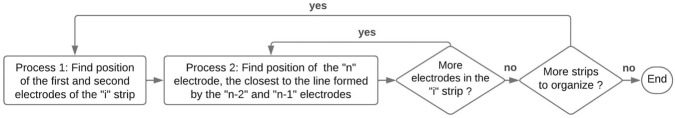
Flowchart outlining the algorithm to automatically label each ECG electrode channel.

The process begins by automatically identifying the first and second electrodes of the first electrode strip. This relies on finding the pixels of the electrode centers in the RGB image with the shortest distance to the first pixel of coordinates (0,0) (Figure 4E). Subsequently, the algorithm identifies the location of the third electrode by searching for the nearest electrode perpendicular to the line formed by the first two electrodes in the (x,y,z) space (Figure 4F). This third electrode is selected if it falls within specific distance criteria: less than 35 mm Euclidean distance and less than 15 mm distance along the x-axis from the second electrode. This process repeats for the remaining electrodes within the same strip, ensuring their optimal placement. The algorithm then proceeds to apply the same procedures to subsequent strips, excluding those electrodes previously labelled. Figure 5 shows this algorithm in flowchart form.

### 2.2 Validation studies

In order to validate our methods for 3D electrode localization, both a phantom study and a clinical study were used.

#### 2.2.1 Phantom validation

A first validation of the system was performed using a phantom torso tank. The 118-channel BSPM electrodes were attached to a human-shaped plastic male torso model (see Figure 1B). The electrode positions were automatically identified with the Intel RealSense Depth Camera D415 and with a smartphone camera to record a video around the phantom torso. The ground truth electrode positions were obtained using a CT scan of the phantom, as well as with an Electromagnetic Tracking System (ETS; Aurora Window Field Generator) as described below.

#### 2.2.2 Clinical validation

The clinical study was approved by the local ethics committee, and written informed consent was obtained from the patient. The 118-channel BSPM electrodes were attached to seven healthy male volunteers (see Figure 1C). Table 1 provides detailed population statistics, including height, weight, age, and BMI for each volunteer.

**TABLE 1:** Population statistics of the seven healthy volunteers.

Healthy volunteer	Gender	Height (cm)	Weight (kg)	Age	BMI
P1	Male	172	58	31	19.6
P2	Male	190	85	24	23.5
P3	Male	183	79	28	23.6
P4	Male	188	75	27	21.2
P5	Male	195	100	29	26.3
P6	Male	174	68	26	22.5
P7	Male	169	65	26	22.8
Average	—	182	76	27	22.8
SD	—	10	14	2	2.1

The volunteers exhibited diverse body shapes, with electrode strips placed at varying distances and orientations to conform to the torso contours of each individual. During the experiments, electrode positions were automatically identified during normal breathing with the Intel RealSense Depth Camera D415 and with a smartphone camera to record a video around the volunteers torso. Ground-truth electrode positions obtained with the ETS as described below.

#### 2.2.3 ECG electrode localization by CTscan

**FIGURE 6:**
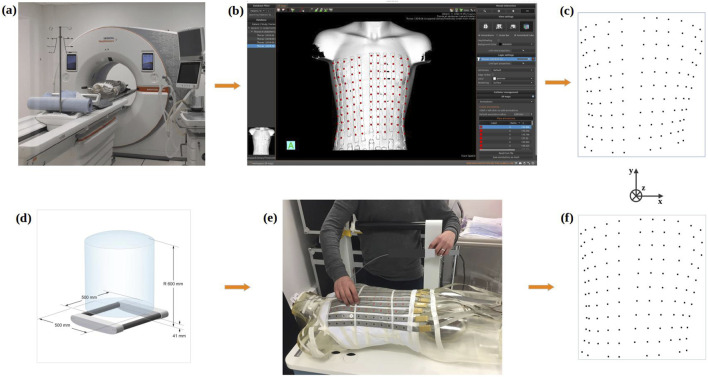
Gold standard methods for ECG electrodes localization process using the CT-scan (top) or an Electromagnetic Tracking System (bottom): **(A)** 3D acquisition of the phantom via CT-scan; **(B)** Reconstruction of the phantom mesh using MusiCardio software; **(C)** Final 3D reconstruction of ECG electrodes from CT-scan; **(D)** Electromagnetic Tracking System and its electromagnetic field generation space; **(E)** Localization of electrodes on the phantom with the Electromagnetic Tracking System; **(F)** Final 3D reconstruction of ECG electrodes from the Electromagnetic Tracking System.

Using the NAEOTOM Alpha® photon-counting CTscan (Figure 6A) (SiemensHealthineers, 2021), a detailed 3D image of the phantom on which the ECG electrodes were placed was generated. The spatial resolution of the CT was 0.11 mm (in-plane), and the slice thickness was 0.2 mm. MUSICardio software (Merle et al., 2022) was employed to reconstruct a 3D torso mesh (Figure 6B) from the CT imaging data. For this reconstruction, a threshold was used to create a mask that isolated the phantom torso with electrode strips from the CT images. This mask was then used to generate the 3D mesh. Subsequently, ECG electrodes were localized and labeled manually on the resulting 3D mesh (Figure 6C), utilizing the depth, width, and height parameters as the Z-axis, X-axis, and Y-axis respectively.

#### 2.2.4 ECG electrode localization by electromagnetic tracking system

The positions of the ECG electrodes were also obtained by employing an Aurora Window Field Generator-type electromagnetic tracking system (Figure 6D). This system was used both with the phantom and for the healthy volunteers. This system uses magnetic sensors to track the movements of a marker emitting a magnetic field (NDI, 2022). To determine the position of ECG electrodes the marker was positioned by hand in the center of each electrode (Figure 6E). To rectify for respiration movement in the volunteers, the marker position was recorded over a short time frame, and the average location found enabling the ECG electrodes to be located in 3D space (Figure 6F). For the phantom, all 118 electrode positions were tagged using the ETS. For the healthy volunteers, 60 electrodes were tagged due to time constraints. In addition, for each healthy volunteer, two ETS markers were fixed to two separate electrodes and their 3D position recorded throughout the procedure.

#### 2.2.5 Localization error metrics

As a measure of error, we considered the Euclidean distance in 3D between the positions of the reconstructed electrodes and those of the reference electrodes, as well as the Euclidean distances along the X-axis, Y-axis, and Z-axis. The results are presented as the mean ± SD unless otherwise stated. Before measuring these distances, we aligned the 3D point clouds of the reconstructed and reference electrodes using the “umeyama” function (Umeyama, 1991). This function finds the transformation parameters that minimize the squared error between two point clouds.

The CT-scan, the ETS and the 3D camera are all calibrated to give distances in the correct scale. This was confirmed by measuring the Euclidean distances between all pairs of electrodes (within every strip) in the point clouds. The systems each produced an average distance of 29.76, 30.04, and 30.32 mm respectively. In contrast, the 2D camera is not calibrated and produces an average distance of 0.027, indicating that this point cloud is in a different scale. To correct the scaling, the known distance of 30 mm was used for the point clouds produced by the 2D camera.

## 3 Results

### 3.1 Phantom data results

**FIGURE 7:**
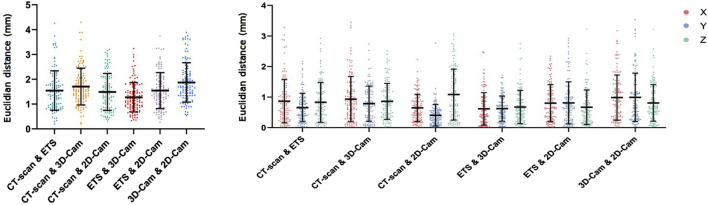
Phantom Data Results: Euclidean distance (mm) between electrodes positions obtained using the CT-scan, electromagnetic tracking system (ETS), 3D camera and 2D camera in 3D (left), and for X, Y, and Z axes (right).

Figure 7 presents a comparison of the 3D Euclidean distances (left) and the Euclidean distances along the X, Y, Z axes (right) between the electrode point clouds obtained for the phantom model with each method. Our comparisons included CT-scan vs. ETS, CT-scan vs. 3D camera, CT-scan vs. 2D camera, ETS vs. 3D camera, ETS vs. 2D camera, and 3D camera vs. 2D camera.

Overall, the mean 3D Euclidean distances were all under 2 mm, suggesting a strong agreement among the different point clouds. The 3D Euclidean distance found between the CT-scan and ETS (1.54 ± 0.79 mm) was comparable to that found between either method and the two camera methods, suggesting the level of accuracy using the 3D visual reconstruction methods is similar to that of current gold standard methods. Likewise, the error was found to be comparable between the 3D camera (1.70 ± 0.74 mm with CT scan or 1.27 ± 0.59 mm with ETS) and the 2D camera (1.49 ± 0.75 mm with CT scan or 1.54 ± 0.72 mm with ETS), meaning neither 3D visual reconstruction method outperformed the other.

The consistency of these results across the different axes (Figure 7 right) suggests that there is no directional bias in the accuracy of the 2D and 3D camera methods. These low values imply minimal variation between the reference methods (CT-scan and ETS) and our two methods (2D camera and 3D camera). Thus, their point clouds are nearly identical.

### 3.2 Clinical data results

**FIGURE 8:**
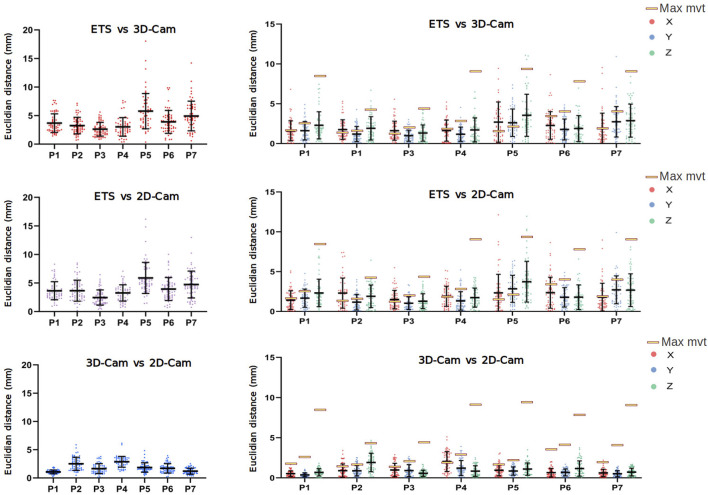
Clinical Data Results: Euclidean distance (mm) between electrode positions obtained using the electromagnetic tracking system (ETS), 3D and 2D camera in 3D (left), and for the X, Y, and Z axes (right). Data obtained from 7 healthy male volunteers (P1 to P7). Yellow rectangles indicate the maximum movement (across X, Y, and Z axes) of two fixed catheters for each patient, as presented in Table 2.

Figure 8 presents a comparison of the 3D Euclidean distances (left) and the Euclidean distances along the X, Y, Z axes (right) between the electrode points clouds obtained with each method on seven healthy volunteers (P1-P7).

Despite the challenges posed by the volunteer’s breathing, the results revealed relatively low error rates. When comparing with the ETS, the 3D camera yielded average 3D Euclidean distances ranging from 2.61 ± 1.2 mm to 5.78 ± 3.09 mm depending on the patient. The 2D camera performed similarly with average Euclidean distances ranging from 2.45 ± 1.32 mm to 5.88 ± 2.73 mm. There was less difference between the 3D and 2D cameras electrode locations with an average 3D Euclidean distances from 1.03 ± 0.39 mm to 2.97 ± 1.08 mm. Consistent with the phantom data results, the analysis of volunteer data shows no consistent directional bias in the accuracy of the 2D and 3D camera methods between the volunteers with results across the X, Y, and Z axes being similar.

**FIGURE 9:**
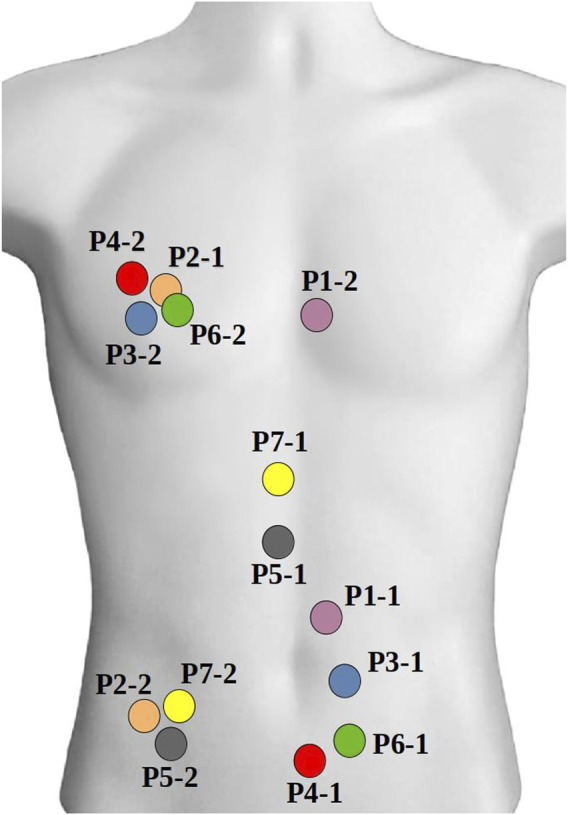
Location of two ETS marker on the torso of healthy volunteers.

Given the variability in error between volunteers, we suspected different respiration patterns may play a role in accuracy. To determine the maximum error in 3D electrode positions one could expect from breathing, we evaluated the degree of respiration movement in each volunteer by tracking the movement of two ETS markers fixed to each volunteer’s torsos. The position of the ETS marker for each volunteer is present in Figure 9 and the maximum movement in along each axis presented in Table 2. For all volunteers, the maximum movement occurred in the z-axis as expected. Interestingly, while large breath movements were associated with larger Euclidean error in two volunteers (P5 and P7), larger respiration movement did not always produce larger errors with 3 volunteers (e.g., P1, P4 and P6) producing comparable error levels to those with relatively little respiration movement (e.g., P2 and P3). The larger error seen with P5 and P7 may then be due to the areas of the chest involved in the respiration movement, that being a mid-chest dominant movement with little lower stomach involvement.

**TABLE 2:** Differences (max - min) in X, Y, and Z coordinates of two ETS markers for the seven healthy volunteers.

Healthy volunteer	ETS marker	X diff (mm)	Y diff (mm)	Z diff (mm)
P1	1	1.42	2.54	8.79
	2	0.92	1.44	2.50
P2	1	1.09	1.44	2.78
	2	1.28	1.54	4.20
P3	1	1.10	1.44	4.34
	2	0.79	2.01	3.75
P4	1	1.86	1.88	9.44
	2	1.87	2.86	5.47
P5	1	1.42	1.45	9.66
	2	1.13	2.14	4.81
P6	1	2.22	3.81	8.38
	2	0.90	4.02	8.10
P7	1	1.83	3.93	9.48
	2	1.86	2.34	6.16

**FIGURE 10:**
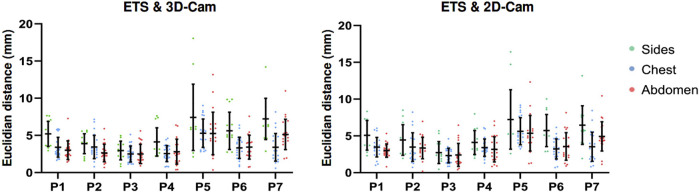
Euclidean distances measured across the chest, abdomen, and torso sides in seven healthy volunteers, for ETS and the 3D camera (left), and ETS with the 2D camera (right).

Regarding the spatial distribution of error on the torso, Figure 10 presents the Euclidean distances measured across the chest, abdomen, and sides for seven healthy volunteers, for ETS and the 3D camera (left), and ETS with the 2D camera (right). The results indicate that errors on the chest and abdomen were generally similar across volunteers, with no significant differences observed between these two regions. However, the sides consistently exhibited marginally larger errors compared to the chest and abdomen across all volunteers, regardless of the method used. Additionally, the similarity between the two graphs demonstrates that the spatial error distribution was comparable between the 2D and 3D camera methods. These findings highlight that the spatial distribution of error on the torso is influenced primarily by the anatomical region and individual respiratory dynamics, rather than the choice of camera system.

Overall, these results validate the effectiveness of both the 2D and 3D camera methods for electrode localization in dynamic clinical conditions and demonstrating satisfactory performance even with breathing-induced variations.

**FIGURE 11:**
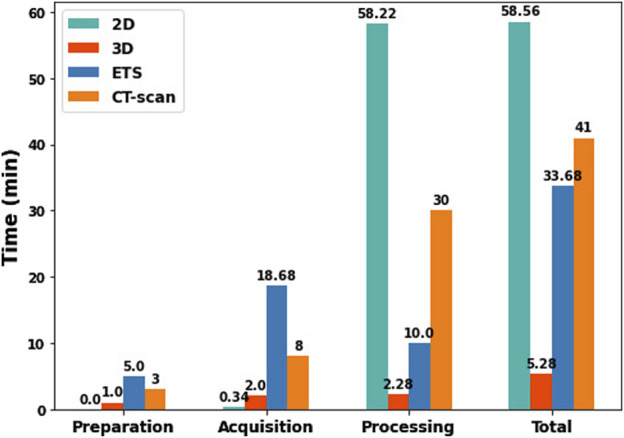
Comparison of preparation, acquisition, processing, and total times between 2D, 3D, ETS, and CT-scan methods.

To compare the time required for each method (2D-camera, 3D-camera, ETS, and CT-scan), we evaluated the time to set up the equipment, data acquisition, data processing, and the total duration for all methods. The times for the 2D, 3D, and ETS methods were based on the average measurements of the seven healthy volunteers, while the times for the CT-scan method were estimated by consulting a radiologist with experience in body surface electrode mapping systems. These results are presented in Figure 11. The 3D method is the most efficient method with a total time to localize the ECG electrodes of 5.28 min including the time to position the camera support behind the bed where the patient is lying and acquire the images at appropriate intervals. The 2D method is the least efficient method, taking nearly an hour, despite being the fastest for preparation (with no equipment preparation required) and acquisition (as the user only needs to record a video of the torso). This is due to the time consuming process of analyzing the video with the Nerfacto model to generate RGB-D images and determine the camera’s intrinsic parameters. The ETS method was the least efficient in terms of preparation (requiring installation of the magnetic sensors around the patients) and acquisition (each electrode needs to be tagged manually). The CT-scan method was fairly inefficient for processing, however in this case the estimation was based on manually segmenting the electrodes. This process and thus the total time could be improved if automated methods were used.

## 4 Discussion

This study presents an innovative and accurate approach for ECG electrode localization using either a 3D DS camera or a 2D video camera. These methods are novel in that they require no additional calibration, or markers to be placed on the electrodes for their automatic detection or labelling, whilst remaining highly competitive by offering comparable performance to previously established methods.

### 4.1 Comparison with previous studies

In the past, several research groups have proposed similar fully or semi-automatic 3D visual reconstruction systems for ECG electrode positioning. Ghanem et al. (2003) were the pioneers, utilizing a two-view 2D camera system with prior camera calibration and manual segmentation to compute the electrodes’ 3D coordinates. Later, Schulze et al. (2014) introduced a similar method but incorporated automatic electrode identification by attaching markers to the electrodes. Both studies reported a mean absolute distance error of approximately 1 mm when using a phantom model.

To overcome the need for camera calibration, more recent studies have used 3D DS cameras (Bayer et al., 2023; Perez-Alday et al., 2018; Shenoy et al., 2024), though continue to use additional markers for automatic electrode identification in the photos. Perez-Alday et al. (2018) were the first to do so using a semi-automated approach. That is, the Kinect 3D camera was used to localize BSPM electrodes by semi-automatically aligning the point clouds to a generic torso mesh and manually selecting the electrode positions. This study reported the 95% limits of agreement exceeded 10 mm for all XYZ coordinates when compared to electrode locations obtained in patients with CT or MRI. More recently, Bayer et al. (2023) and Shenoy et al. (2024) introduced fully automated methods for electrode localization from 3D DS images, though neither study has extended this to automatic labeling of electrodes after detection. Bayer et al. (2023) used simple image processing techniques to automatically segment and identify the electrodes based on their shapes. With these techniques they achieve an average positional deviation of 2.0 ± 1.5 mm from markers placed manually on the same 3D images. While Shenoy et al. (2024) did not detail the specific methods for electrode detection, they did compare the electrode positions obtained to CT scan-based electrode locations in patients, demonstrating their system has an average localization error of 50.2 mm. The majority of this larger average error is likely due the change in posture of the patient between the supine recordings in the CT and upright position used with the 3D camera.

In this study we have presented two novel methods that have improved the workflow of these previous established methods using Artificial Intelligence (AI), whilst still offering a similar level of high precision with average localization errors less than 2 mm when using a phantom model. Furthermore, while this study presents an application for the BioSemi BSPM lead set, with further training the methods could easily be adapted to allow fully automatic segmentation of any BSPM lead set.

For the first method using a single 2D camera, we have removed the need for camera calibration by using the Nerfacto deep neural network architecture to generate the camera’s intrinsic parameters and the depth images. Whilst this method offers the benefit of being used with any 2D camera, the use of the Nerfacto does substantially increase the computational time taking approximately 1 h to run for one patient. The computational time for Nerfacto depends directly on the number of frames of the video taken. This time could be improved by down sampling the video, however this may also impact the accuracy of the resulting RGB-D images and intrinsic camera parameters, ultimately impacting the 3D location of the electrodes.

The second method we presented using a 3D DS camera stands out for its rapid acquisition requiring 5 min to obtain accurate measurements. This difference in acquisition time highlights the practical advantage of the 3D DS method in clinical settings where time is a critical factor. For both methods, we have also eliminated the need for additional markers for electrode identification by using a YOLOv8 model trained to automatically detect the electrodes themselves. By not requiring physical markers for electrode detection, the procedure is simplified and reduces the risk of marker-related errors.

### 4.2 Impact of respiration

One of the key challenges in localizing ECG electrodes is the movement of the patients torso during acquisition, in particular with respiration. Our clinical validation has demonstrated that our methods are well-suited for use in dynamic and real-world conditions, providing satisfactory precision even with bodily movement (localization error ranging from 2.45 to 5.88 mm). For use in an ECGI pipeline, this level of error is well within the acceptable range that has been shown to yield satisfactory results in previous studies (Jiang, 2010; Cheng, 2001; Burnes et al., 2000a; Burnes et al., 2000b). To enhance our approach, multiple cameras could be deployed around the torso to capture images simultaneously. This would allow us to obtain the 3D electrode positions during any phase of the respiration cycle, potentially improve alignment accuracy and reduce acquisition time.

### 4.3 Decision making for choice of method

The choice of method depends on the available resources, time efficiency, and user training requirements. Below we outline these for both presented methods:

#### 4.3.1 Equipment

The 3D method requires specific equipment including a depth sensing camera (such as the Intel RealSense, priced between 200 and 500$), a support structure to hold and rotate the camera around the torso (e.g., a tripod with an articulated arm, costing an additional 100 to 200$), and a standard computer. While the 2D method only requires more standard equipment; any 2D video camera (such as a smartphone) and a computer, the computer does require a GPU of at least 4 GB of memory (starting at 500$) to process data using the Nerfacto model. A web interface could be developed for the 2D method, enabling users to upload videos of the torso with ECG electrodes and automatically retrieve the electrode positions. In this case, users would not need a computer with a GPU, as the processing could be done remotely via the web interface.

#### 4.3.2 Time

In terms of time efficiency, the 3D method is significantly faster, taking only 5 min to complete the entire process, including preparation of equipment, data acquisition, and processing. In contrast, the 2D method requires much more time, an average of 60 min, mainly due to the time consuming processing involved with the Nerfacto model (Figure 11).

#### 4.3.3 User training

Both the 3D and 2D camera methods require minimal training. For the 3D method, typically 5–10 min is enough to ensure the user can operate the system effectively including mounting support structure behind the bed, ensuring the depth sensing camera is properly aligned approximately 60–70 cm above the patient’s chest. Once the setup is complete, the user launches the script, which provides real-time visualization of the detected electrodes. The acquisition process involves capturing 12 RGB-D images from predefined positions around the torso. The camera support includes visual markers indicating each of the 12 stops. After capturing all 12 images, the system then automatically processes the data, localizes the electrodes, and saves all results in a specified folder. In the future, the 3D method could be further optimized by developing a system with three 3D cameras positioned around the torso. This setup would eliminate the need to rotate a single camera, allowing for instant data capture. Such a configuration could minimize errors caused by patient breathing or motion during the process.

For the 2D camera method, the user only needs to record a high-quality video of the torso using a smartphone. This video is then provided as input to the script, which processes the data over approximately 1 h to localize the electrodes and save the results in a specified folder. To further simplify the installation and use of both methods, all scripts and required libraries could be hosted on a GitLab repository. Users would have centralized access to the entire workflow, including detailed documentation. Additionally, to streamline setup and compatibility, all necessary components (scripts, dependencies, and libraries) could be packaged within a Docker container. This approach ensures that users could deploy the system effortlessly, regardless of their local operating environment, while minimizing potential configuration errors.

Overall, the 3D method is faster but requires very specific equipment. In comparison, the 2D method is simpler, needing only a smartphone and a computer with good GPU, with the option of a web interface for even easier access. User training for both methods is simple and requires minimal time. The final choice thus depends on the specific clinical context: the 3D method is ideal for situations where speed is critical and the resources are available, while the 2D method is a practical option when simpler equipment or remote processing is preferred.

### 4.4 Generalization for other electrode systems

Our methods can be adapted to other electrode systems beyond the BioSemi strips used in this study. For most systems, our methods remain applicable with some adjustments. Specifically, if the electrodes differ, a detection and classification model would need to be retrained to recognize the new electrode color/shape scheme. To organize the detected electrodes, our organization algorithm could still be applied, provided the electrodes are in strips and specific characteristics, such as inter-electrode distances, are taken into account. Our method has demonstrated robustness in handling tilted electrode strips or strips placed with varying spacings, as supported by the clinical validation.

For systems without a strip like structure, such as the vest-based Corify and CardioInsight systems, our approach could be implemented by retraining the deep learning model to detect the new electrode shapes or configurations, with an adjusted ECG electrode organization algorithm to accommodate the new orientations or structural layouts. In certain cases, the organization algorithm may be uneccesary. For example, in the case of CardioInsight, a deep learning model could be trained to detect regions corresponding to ECG electrodes, followed by an image processing algorithm or another deep learning model to identify and classify the numbers associated with each region. For Corify, the process is even simpler: ECG regions can be identified using ArUco markers, which can be reliably detected with the OpenCV library, as each marker has a unique code. This enables precise localization of the electrodes without relying on a deep learning model or a specific organization algorithm.

## 5 Limitation

Despite the promising results, several limitations should be acknowledged. Firstly, the process of aligning the point clouds obtained from CT, ETS and the two camera methods relied on the electrode positions themselves. Ideally an independent marker should have been used for this alignment to avoid underestimating the localization error.

Furthermore, although the reference methods used are considered gold standard, they can produce their own errors in localization of around 1 mm. For CT, manual selection of electrodes within the mesh may introduce errors. Similarly, for ETS, manual positioning of electrodes with a catheter can also contribute to inaccuracies.

Regarding the 2D camera method utilizing the Nerfacto model, it requires a GPU with a minimum of 4 GB of memory to ensure adequate processing performance. Additionally, the video of the torso with the electrodes captured by the 2D camera should be lightweight (less than 15 MB for a GPU with 4 GB memory) while maintaining high quality to enable accurate 3D reconstruction of the electrodes.

For the YOLOv8 based electrode detection, maintaining a well-lit environment with clear white lighting is essential. Proper lighting allows the camera to accurately capture the true colors of the electrodes, which is critical for the model to differentiate between electrode classes effectively. Inadequate lighting conditions or colored light sources could lead to misclassification or detection errors.

Lastly, the study was only validated on relatively healthy males with low BMI, which limits the generalizability of the findings. Future studies should expand the clinical evaluation to include women, individuals with higher BMI, and other diverse patient populations.

## 6 Conclusion

This study introduces two innovative methods for accurate ECG electrode localization: a 3D Depth Sensing (DS) camera-based method and a 2D camera-based method. Both methods demonstrated exceptional precision, with localization errors from 2 to 5 mm in patients.

The 3D DS camera method provides rapid and precise measurements within 5 min, making it ideal for clinical settings where time efficiency is crucial. Meanwhile, the 2D camera method, though more time-consuming, also achieves high precision and is effective for scenarios where time constraints are less critical and specific 3D DS cameras are not available.

These methods offer effective alternatives to traditional imaging techniques such as CT scans and MRI. They not only enable accurate and efficient electrode localization but also address practical challenges associated with conventional imaging. This could have significant implications for clinical practice, particularly in scenarios where traditional imaging methods are impractical or contraindicated, such as in healthy volunteers for clinical studies or in patients with metallic implants. By facilitating the broader adoption of advanced ECG mapping techniques, these methods represent a meaningful advancement in clinical practice.

## Data Availability

The original contributions presented in the study are included in the article/supplementary material, further inquiries can be directed to the corresponding author.
